# Dynamic Anterior Shoulder Stabilization Using a Long Head of the Biceps Transfer and Bankart Repair Improves Clinical Outcomes in Patients With Subcritical Bone Loss: A Systematic Review

**DOI:** 10.1016/j.asmr.2025.101141

**Published:** 2025-04-10

**Authors:** Neil Jain, Jonathan McKeeman, Margaret Higgins, Alexander Johnson, Tyler Smith, Brian Waterman

**Affiliations:** a Department of Orthopaedic Surgery, St. Luke’s University Health Network, Bethlehem, Pennsylvania, U.S.A.; b Department of Orthopedic Surgery, Atrium Health Wake Forest Baptist Orthopaedics and Sports Medicine, Winston-Salem, North Carolina, U.S.A.

## Abstract

**Purpose:**

To assess the clinical outcomes of dynamic anterior stabilization using a long head of the biceps transfer (DAS-LHB) with Bankart repair for management of anterior shoulder instability with subcritical glenoid bone loss.

**Methods:**

Using the Preferred Reporting Items for Systematic Reviews and Meta-Analyses guidelines, a review of the PubMed, Scopus, EMBASE, and Medline databases was performed in March 2025. Studies were limited to those reporting outcomes for patients ≥16 years old with subcritical glenoid bone loss (≤20%) and undergoing primary DAS-LHB with Bankart repair. Articles were assessed for range of motion, patient-reported outcomes, recurrent instability, and reoperation rates. The Methodological Index for Non-Randomized Studies scoring criteria were used for study appraisal. Forest plots were created to visualize mean differences in variables of interest.

**Results:**

Among the 4 studies evaluated, 90 patients with mean ages of 23.4 to 31.9 years were included. Average follow-up and bone loss ranged from 24.0 to 41.8 months and 8.0% to 10.5%, respectively. Rowe scores showed improvements ranging from 54.5 to 74.0 points, with 87.9% to 100% of patients meeting minimal clinically important difference thresholds. Changes in forward flexion and external rotation ranged from 3° to 23° and –2.3° to 8.3°, respectively. Four patients (4.4%) experienced a recurrence of dislocation, of whom 2 underwent successful revision with a Latarjet procedure. Of all patients, 90.1% to 100.0% were able to return to sport at any level, while 60.0% to 78.8% returned to their preinjury level of athletic involvement.

**Conclusions:**

DAS-LHB in conjunction with Bankart repair preserves motion while showing clinically important functional improvements with low rates of recurrent instability or complication in the short-term postoperative period for patients with symptomatic shoulder instability.

**Level of Evidence:**

Level IV, systematic review of Level III and IV studies.

Many techniques are available for surgical management of anterior shoulder instability (ASI). Soft tissue procedures such as Bankart repair, capsulorrhaphy, and remplissage have satisfactory clinical outcomes, yet a high rate of recurrent instability is observed among contact athletes and high-risk patients with humeral and/or glenoid bone loss.^1^, ^2^, ^3^, ^4^, ^5^, ^6^, ^7^, ^8^, ^9^ Conversely, coracoid autograft transfer via the Latarjet procedure is considered the gold standard for bone loss exceeding a critical value of 20%.^7^ This threshold has recently been redefined at 13.5%, and there has been an increasing trend in the use of Latarjet and free bone block (FBB) procedures.^7^ While these techniques have a low risk of recurrent instability, difficulties associated with their use include a steep learning curve, risk of intraoperative neurovascular injury, graft nonunion, and hardware-related complications.^1^^,^^6^^,^^10^, ^11^, ^12^, ^13^, ^14^, ^15^, ^16^, ^17^

For patients with engaging Hill-Sachs lesions and “subcritical” bone loss in North America, arthroscopic Bankart repair with remplissage has largely been preferred over a Latarjet procedure given its comparable outcomes and fewer overall postoperative complications.^18^, ^19^, ^20^ However, limitations in rotational range of motion (internal-external) and revision rates ranging from 2% to 3% may not be optimal for overhead athletes.^18^, ^19^, ^20^, ^21^ Recently, dynamic anterior stabilization (DAS) has emerged as an option for bridging the gap between soft tissue and bony procedures. In these instances of subcritical bone loss, a tendon sling effect is achieved through creation of a subscapularis split and transfer of either the conjoint tendon (DAS-CT) or long head of the biceps (DAS-LHB) onto the anterior glenoid rim.^2^^,^^6^^,^^22^, ^23^, ^24^, ^25^ When compared to preoperative baselines, this has resulted in satisfactory clinical outcomes and low rates of recurrent instability. Furthermore, DAS has the potential advantages of safer and easier surgical technique, lower risk of neurologic injury, and decreased risk of hardware-related complications. While conjoint tendon transfer has been well characterized in the literature, results from using the long head of the biceps are not well understood.

Therefore, the purpose of this study was to assess the clinical outcomes of dynamic anterior stabilization using a long head of the biceps transfer with Bankart repair for management of anterior shoulder instability with subcritical glenoid bone loss.

## Methods

### Literature Search and Study Eligibility

A systematic review was registered in PROSPERO and performed following the Preferred Reporting Items for Systematic Reviews and Meta-Analyses guidelines. A literature search of the PubMed, Scopus, Embase (Elsevier), and Medline (Ovid) databases was completed for articles discussing DAS on March 5, 2025.^26^ A search strategy was devised using key phrases to ensure a complete collection of articles (Supplement 1). After completion of the literature search, citations from included studies were cross-referenced to identify articles that may have been missed.

Studies were included if they reported clinical outcomes for patients ≥16 years old with subcritical glenoid bone loss undergoing primary DAS-LHB and Bankart repair to manage anterior shoulder instability. Subcritical bone loss was defined as ≤20% as determined on computed tomography or magnetic resonance imaging. Exclusion criteria were: lack of relevance to DAS, technical notes, biomechanical studies, case series with fewer than 3 patients, review/background studies, conference abstracts, editorial commentaries, no full-text availability in the English language, lack of outcome data compared to a preoperative status, inadequate (<12 months) clinical follow-up, use of DAS in isolation or with procedures other than Bankart repair, and articles of “very low” or “low” quality as determined during the appraisal process.

### Screening, Data Abstraction, and Variables Included

A full-text review to confirm appropriateness for inclusion was independently completed by 2 authors (J.M. and M.H.). A κ score of 0.738 showed substantial agreement among reviewers. Any disputes during the screening process were resolved by discussion with a third author (T.S.) assisting with final article selection.

Studies meeting inclusion criteria were moved to data abstraction. Two authors (J.M. and M.H.) independently extracted the following information into datasheets: author, year of publication, journal name, number of DAS-LHB patients, mean follow-up (months), mean patient age (years), mean glenoid bone loss (%), methodology used for estimation of glenoid bone loss, and study design. The primary outcomes examined were Rowe scores, range of motion (ROM), return to sports at any level, return to sports at the preinjury level, recurrence of instability events, and reoperation rates.

### Risk-of-Bias Assessment

Risk of publication bias and study quality were appraised using the Methodological Index for Non-Randomized Studies (MINORS) scoring criteria.^27^ This validated instrument has 8 or 12 items depending upon a study’s noncomparative or comparative design. The maximum scores for either are 16 and 24, respectively. For noncomparative studies, interpretation of the overall score is as follows: 0 to 4, very low quality; 5 to 8, low quality; 9 to 12, moderate quality; and 13 to 16, high quality.^28^^,^^29^ For comparative studies, scoring is as follows: 0 to 6, very low quality; 7 to 12, low quality; 13 to 18, moderate quality; and 19 to 24, high quality.^28^^,^^29^ A detailed breakdown of MINORS scoring is provided in Table 1. Article level of evidence was determined using guidelines published by the *Journal of Bone and Joint Surgery* in 2015.^33^ An aggregate assessment of included study demographics, methodology, and scoring is in Table 2.

**Table 1:** Methodological Index for Non-Randomized Studies Scoring After Study Quality Appraisal

Study	Collin et al.,^30^ 2022	de Campos Azevedo et al.,^9^ 2023	de Campos Azevedo et al.,^31^ 2021	Garcia et al.,^32^ 2024	Wu et al.,^2^ 2023
1. Clearly stated aim	2	2	2	2	2
2. Inclusion of consecutive patients	2	1	1	2	2
3. Prospective collection of data	0	2	0	2	0
4. Endpoints appropriate to the aim of the study	2	2	2	2	2
5. Unbiased assessment of the study endpoint	0	0	0	0	0
6. Follow-up period appropriate to the aim of the study	2	2	2	2	2
7. Loss to follow-up less than 5%	2	2	0	2	2
8. Prospective calculation of the study size	0	2	0	1	2
**Additional criteria for comparative studies**					
9. An adequate control group	—	—	—	—	2
10. Contemporary groups	—	—	—	—	2
11. Baseline equivalence of groups	—	—	—	—	2
12. Adequate statistical analyses	—	—	—	—	2
Total score	10/16	13/16	7/16	13/16	20/24
Study quality	Moderate	High	Low	High	High
Level of evidence	IV	IV	IV	IV	III

**Table 2:** Study Demographics, Methodology, and Scoring

Author	Year	Journal	Number of DAS-LHB Patients	Mean Follow-Up, mo	Mean Age, y	Mean Glenoid Bone Loss (%)	Methodology/Design	Level of Evidence	MINORS Score
Collin et al.^30^	2022	*Arthroscopy*	22	38.4	31.9	9.0	Retrospective case series	IV	10/16
							No control group		
							No power analysis completed		
de Campos Azevedo et al.^9^	2023	*ASMR*	15	23.93	23.4	8.2	Prospective case series	IV	13/16
							No control group		
							Power analysis completed for subgroup analysis		
Garcia et al.^32^	2024	*JSES International*	20	>24	30.95	8.0	Prospective case series	IV	13/16
							No control group		
							Power analysis completed		
Wu et al.^2^	2023	*Arthroscopy*	33	41.8	26.3	10.5	Retrospective comparative cohort study	III	20/24
							Compared DAS-LHB to DAS-CT		
							Power analysis completed		

DAS-CT, dynamic anterior stabilization using a conjoint tendon transfer; DAS-LHB, dynamic anterior stabilization using a long head of the biceps transfer; MINORS, Methodological Index for Non-Randomized Studies.

### Statistical Analysis

R software version 4.4.1 (R Foundation for Statistical Computing) was used to create forest plots and visualize the changes in Rowe scores, forward flexion (FF), and external rotation (ER). A meta-analysis was not performed due to low article level of evidence (III, IV) and heterogeneity between studies. Outcomes for variables of interest were presented as a qualitative synthesis of findings. Ranges were included where appropriate.

## Results

### Systematic Review

**Fig 1:**
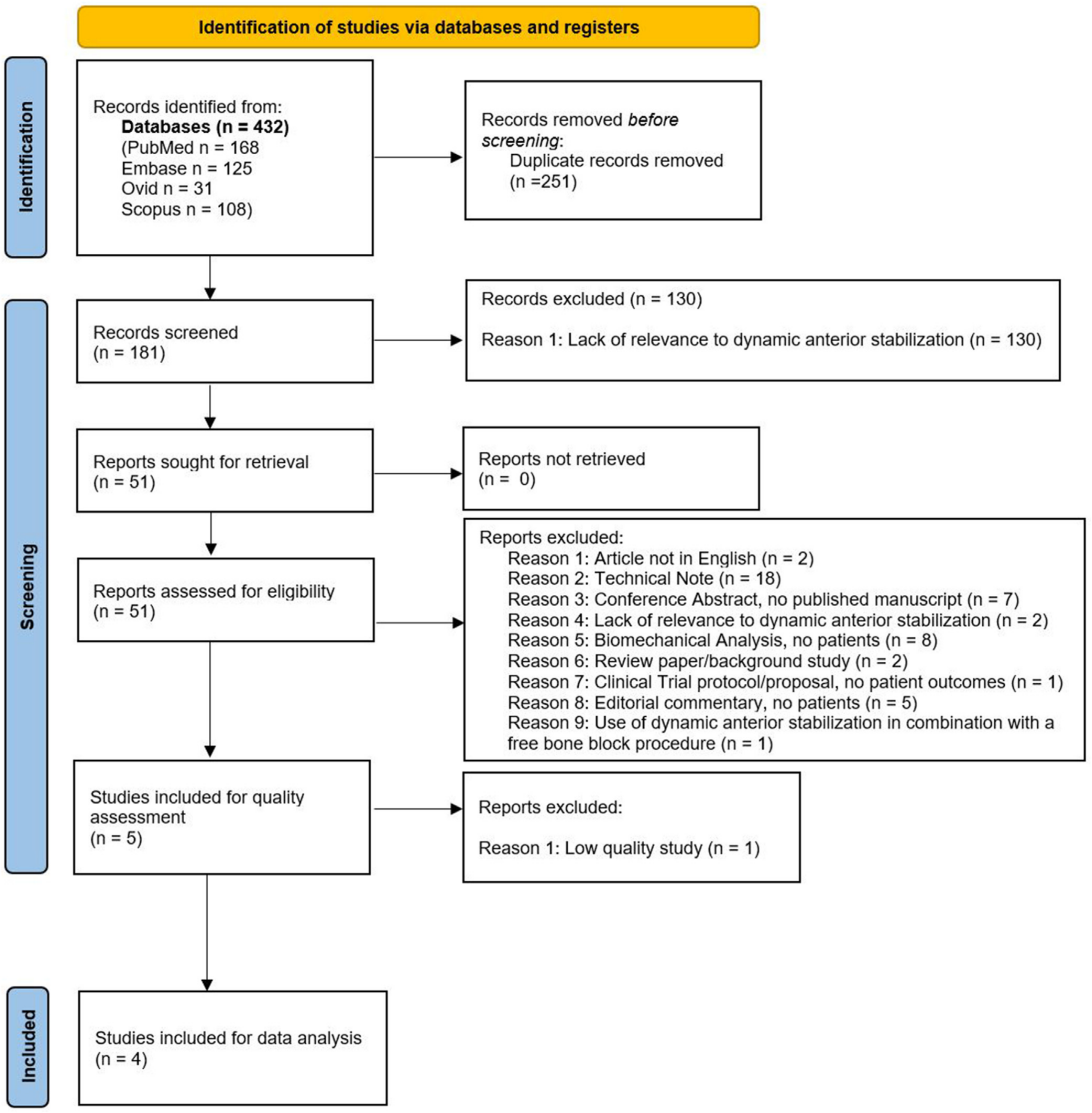
Preferred Reporting Items for Systematic Reviews and Meta-Analyses diagram illustrating the study selection process.

A Preferred Reporting Items for Systematic Reviews and Meta-Analyses flow diagram outlining the study identification, selection, and screening process can be found in Figure 1. After quality assessment using the MINORS scoring criteria, 1 low-quality article^31^ was excluded. The remaining 4 articles^2^^,^^9^^,^^30^^,^^32^ were included for data analysis.

### Surgical Techniques

All studies followed previously published techniques describing DAS-LHB.^22^^,^^23^^,^^34^^,^^35^ An arthroscopic approach with concurrent Bankart repair or capsulorrhaphy was common to all methodologies. No concurrent remplissage was performed. A biceps tenotomy was performed in all studies, but 1 article^9^ used a novel double double-pulley (DDP) technique and did not require braiding the proximal tendon before attachment to the anterior glenoid margin. Graft fixation to the glenoid varied widely between studies. Two articles^2^^,^^32^ passed a cortical button complex through a transverse glenoid tunnel, and 1 study^30^ used a drill hole for placement of an interference screw. Authors utilizing the DDP technique completed an onlay DAS-LHB using suture anchors only.^9^

### Study Characteristics and Bias Assessment

Table 2 summarizes study characteristics. Among the 4 included articles, 2 were retrospective reviews^2^^,^^30^ and 2 were prospective case series.^9^^,^^32^ Ninety patients received DAS-LHB, of whom 70 (77.8%) were male. Individuals had mean glenoid bone loss ranging from 8.0% to 10.5%. These estimations were determined using computed tomography in 2 studies,^2^^,^^30^ while the remainder^9^^,^^32^ utilized magnetic resonance imaging.

Both prospective studies included an a priori sample size calculation and were adequately powered. One retrospective cohort study^2^ compared outcomes of DAS-LHB to DAS-CT. Some validated tools used to report clinical outcomes differed between studies and were not included for data analysis. A summary of these results is in Table 3. The included articles contained Level III or IV evidence. The mean MINORS score for 3 articles^9^^,^^30^^,^^32^ assessed using the 16-point scale was 12.0. The fourth article^2^ scored a 20.0 on the 24-point scale.

**Table 3:** Summary of the Clinical Outcome Measures Not Included in Data Analysis

Author	Year	Journal	American Shoulder and Elbow Surgeons Score		Oxford Shoulder Instability Score		University of California–Los Angeles Score		Western Ontario Shoulder Instability Index	
			Preop	Postop	Preop	Postop	Preop	Postop	Preop	Postop
Collin et al.^30^	2022	*Arthroscopy*	—	—	—	—	—	—	—	—
de Campos Azevedo et al.^9^	2023	*ASMR*	—	—	—	—	—	—	1,302.9 ± 309.8	343.7 ± 376.7
Garcia et al.^32^	2024	*JSES International*	85.0 ± 8.9	97.3 ± 4.4	—	—	25.6 ± 2.8	34.6 ± 0.8	—	—
Wu et al.^2^	2023	*Arthroscopy*	73.8 ± 21.3	95.0 ± 8.8	35.2 ± 9.4	14.8 ± 2.8	—	—	—	—

### Clinical Outcomes and Complications

##### Rowe Scores

**Fig 2:**
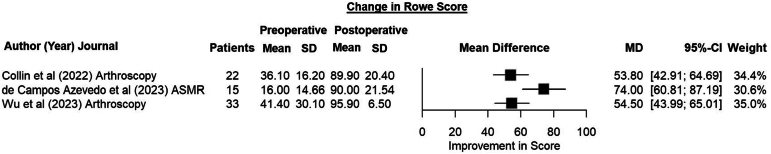
Forest plot showing the improvement in Rowe scores after dynamic anterior stabilization using a long head of the biceps transfer with Bankart repair. (CI, confidence interval; MD, mean difference; SD, standard deviation.)

All studies reported improvements in Rowe scores measured perioperatively. Three articles^2^^,^^9^^,^^30^ included preoperative means and noted improvements ranging from 54.5 to 74.0 points (Fig 2). The highest score increases were shown in patients undergoing DAS-LHB using a DDP technique.^9^ Two studies^9^^,^^30^ observed 91% to 100% of patients improving beyond a minimal clinically important difference threshold of 9.7 points. Wu et al.^2^ used a minimal clinically important difference of 13.2 and reported 87.9% of patients as meeting or exceeding this value. However, this finding was not significant when compared to the DAS-CT cohort (87.9% vs 93.3%, *P* = .674, respectively).^2^ One study^32^ defined Rowe scores above 90 as indicative of excellent postoperative results and showed statistically significant outcomes when compared to this standard (*P* < .01).

##### Range of Motion

**Fig 3:**
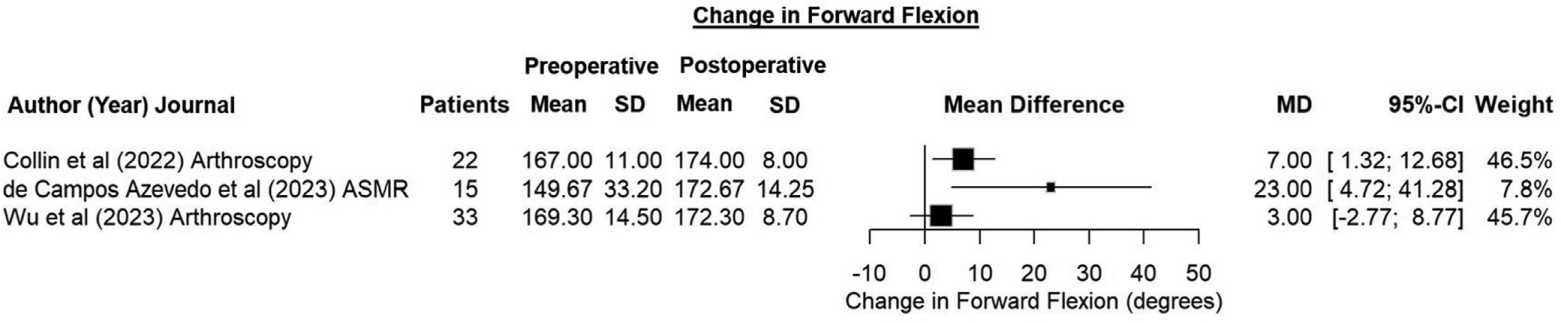
Forest plot showing the changes in forward flexion after dynamic anterior stabilization using a long head of the biceps transfer with Bankart repair. (CI, confidence interval; MD, mean difference; SD, standard deviation.)

**Fig 4:**
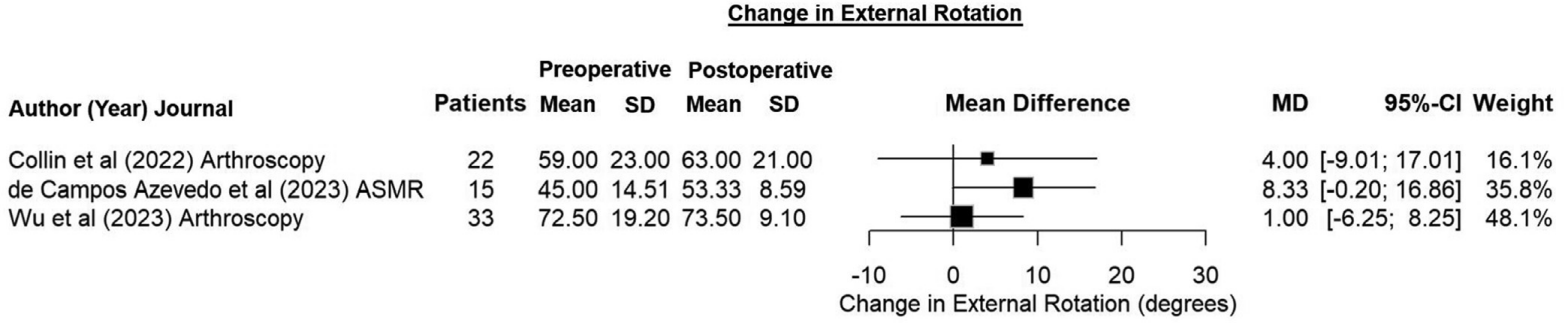
Forest plot showing the changes in external rotation after dynamic anterior stabilization using a long head of the biceps transfer with Bankart repair. (CI, confidence interval; MD, mean difference; SD, standard deviation.)

Three studies^2^^,^^9^^,^^30^ reported improvements in FF ranging from 3.0° to 23.0° (Fig 3). All studies included ER data, but only 3^2,9,33^ reported preoperative means. The average improvements in ER observed by these studies ranged from 1.0° to 8.3° (Fig 4). In contrast, Garcia et al.^32^ observed an average loss of 2.3° in their series. DDP patients had the largest increases in both FF and ER.^9^ Three studies^2^^,^^9^^,^^30^ measured changes in internal rotation by estimating the highest vertebral body that the thumb of the measured arm could reach. Using this metric, changes in internal rotation levels ranged from –1.0 to 0.74.

##### Return to Sports

Three studies^2^^,^^9^^,^^32^ reported overall rates of return to sports. Of these 68 patients, 90.1% to 100.0% were able to return to sport at any level, while 60.0% to 78.8% returned to their preinjury level of athletic involvement. DDP patients were observed to have the lowest rate of return to preinjury athletics (60%).^9^ When comparing DAS-LHB and DAS-CT cohorts, Wu et al.^2^ noted no significant differences in rates of return to sport at any level (90.1% vs 86.7%, *P* = .700, respectively). These findings remained unchanged when examining rates of return to preinjury level of athletic involvement (78.8% vs 73.3%, *P* = .258, respectively).^2^

Two studies^2^^,^^32^ included competitive or contact athletes as part of their inclusion criteria but did not provide specific subgroup data for these populations. Of the 4 (26.7%) DDP patients participating in competitive sports, all were able to return to play.^9^

##### Complications/Reoperations/Donor Morbidity

Four patients (4.4%) experienced a recurrence of instability.^9^^,^^30^ One study^30^ reported 3 of these complications and successfully revised 2 patients with a Latarjet procedure. The authors attributed the dislocation rate in their series to the learning curve associated with DAS-LHB.^30^ It was suggested that dislocation may have recurred due to use of interference screws with an inadequate diameter or an insufficient long head of the biceps (LHB) length during introduction into the glenoid.^30^ No infections, neurovascular injuries, Popeye deformities, muscular cramps, or postoperative subscapularis dysfunction were reported in the included studies.

## Discussion

The findings of the present review show that dynamic anterior stabilization is an acceptable procedure for patients with anterior shoulder instability and subcritical bone loss. Improvements in Rowe scores indicated that nearly all patients met minimal clinically important difference thresholds without losses in ROM. Only a small subset of patients experienced recurrent instability, which was significantly lower than the published rates for recurrent instability among Bankart repair with and without remplissage.^36^, ^37^, ^38^, ^39^ As such, DAS-LHB may serve as a valuable surgical option to serve as an adjunct to Bankart repair and to potentially avoid bone block procedures.

### DAS-LHB Versus DAS-CT

Although prior descriptions of dynamic anterior stabilization have included transfer of the conjoint tendon, recent literature has trended toward use of the long head of the biceps. In the technical note of Collin and Lӓdermann,^22^ DAS-LHB was described as including concomitant Bankart repair to restore normal articular concavity. Cadaveric biomechanical studies conducted on shoulders with 13% and 20% glenoid bone loss showed this combination to be significantly stronger than capsulolabral repair in isolation or with conjoint tendon transfer augmentation.^40^^,^^41^ However, these findings have not been reflected in clinical studies. Wu et al.^2^ examined outcomes of patients with ASI and subcritical bone loss who were treated with DAS-LHB or DAS-CT. Both cohorts had similar functional scores and comparable low rates of recurrent dislocation.^2^ Their results dismissed concerns that the smaller diameter of the LHB tendon contributes to an inferior tendon sling effect. Furthermore, Douoguih et al.^25^ reported on a series of 10 patients with critical (>25%) glenoid bone loss treated with DAS-CT. Of those available for follow-up, only 1 experienced recurrent instability.^25^ Given these successful outcomes, future studies are warranted to determine if DAS-LHB is a treatment option for patients with critical bone loss.

Morbidity associated with transfer of the conjoint tendon may be an important determinant in surgical decision-making. In comparison to arthroscopic LHB tenotomy, harvest of the conjoint tendon done in a similar manner is a time-consuming and more technically demanding process.^2^ If an open technique is used, the larger incision required for the deltopectoral approach could lead to soft tissue and cosmetic defects, as well as disruptions to operative workflow.^2^ Preservation of the coracoid in an LHB transfer leaves the pectoralis minor intact and maintains the working length of the coracobrachialis and short head of the biceps.^2^^,^^22^^,^^42^ This lower requirement for surgical manipulation may theoretically help reduce the risk of scapular dyskinesis and, potentially, neurovascular injury.^2^^,^^22^^,^^42^ In a case report by DeFroda et al.,^43^ DAS-LHB was used to successfully manage a patient with musculocutaneous nerve palsy and recurrent dislocation after Bankart repair. Techniques that involve transfer of the coracoid or conjoint tendon were contraindicated due to the associated risk of placing traction on the nerve.^43^ At a 16-month follow-up, no additional instability events were noted. These advantages suggest DAS-LHB may incur fewer complications in the short term, but the lack of high-level evidence and long-term follow-up makes it difficult to draw definitive conclusions between the 2 techniques.

### DAS-LHB Versus Latarjet

Indications for a Latarjet procedure include ASI with critical glenoid bone loss or significant risk factors for recurrent dislocation, such as participation in contact sports, bipolar bone loss, or failed arthroscopic stabilization procedures.^17^ While DAS-LHB has been traditionally indicated for subcritical bone loss, Douoguih et al.^25^ reported satisfactory outcomes for conjoint tendon transfers done for bone loss >25%. For this subset of patients, DAS-LHB may be similarly successful and potentially advantageous due to its lower complication rates. In their review of 72 articles, Arner et al.^44^ found that posttraumatic osteoarthritis, graft osteolysis, nonunion, neurovascular injury, and hardware complications were among the most frequently encountered issues after a Latarjet procedure. Additional systematic reviews and meta-analyses have corroborated these findings, with rates of reoperation ranging from 2.6% to 7%.^14^^,^^16^^,^^17^^,^^44^ In the present analysis, no studies reported nerve injury, fracture, infection, or hardware problems related to DAS-LHB. Recently published technical modifications have suggested augmenting biceps tendon transfer with anchorless or all-suture fixation, and this may further lower the risk of infection or hardware-related complications well below what is seen with the Latarjet.^9^^,^^34^^,^^45^

### DAS-LHB as an Adjunct

Given its simple technical requirements, DAS-LHB may be indicated as a supplement for a wide range of procedures. In this review, a total of 4 (4.4%) patients experienced recurrent instability events, with 3 reported from a single study.^30^ Two went on to successful revision with a Latarjet, but the authors did not comment on the difficulty of doing so.^30^ In theory, the interference screw used to secure the LHB onto the glenoid rim could alter the technical considerations of the procedure, including risk of fracture, increased bone loss, and nonunion or fibrous union. In addition, revision exposure could be more challenging with a prior dissection through the subscapularis.^46^ Regardless, the 13.6% recurrent dislocation rate reported by Collin et al.^30^ is still lower than that of isolated Bankart repair when subcritical glenoid bone loss is present.^7^

Several FBB techniques have gained popularity as alternatives to the Latarjet procedure. Although many autograft and allograft options have shown promise for the management of glenoid bone loss, the safety and efficacy of iliac crest and distal tibia grafts are the most frequently described.^47^ In the report by Wu et al.,^1^ external rotation restrictions were observed after DAS-LHB was combined with an allogenic iliac crest FBB. When compared to clinical studies without a concomitant FBB procedure, it is unlikely that ROM restrictions were caused by DAS-LHB. Mehl et al.^6^ completed a biomechanical cadaveric study on 24 shoulders and examined the stabilizing effects of DAS-LHB versus standard Bankart repair. When compared to the native shoulder, DAS-LHB was found to preserve rotational ROM.^6^ The data in our study revealed these findings, with 1 article indicating significant improvements in forward flexion and external rotation if an onlay DAS-LHB is completed using a DDP technique.^9^ This maintenance of motion may be particularly useful for overhead athletes and implicates wider use of DAS-LHB before bony reconstruction is considered. Arthroscopic Bankart repair with remplissage and Latarjet procedures cause considerable changes to motion and are likely to significantly impact the career for throwers. As a bridge between soft tissue and bony procedures, DAS-LHB may be an optimal choice for athletes if subcritical bone loss is present.

### Return to Sports

Our review found athletes returned to sports at a high rate after DAS-LHB. Of the patients, 90.1% to 100.0% were able to return to sport at any level, while 60.0% to 78.8% returned to their prior athletic intensity. When compared to standard Bankart repair, remplissage, and Latarjet procedures, these results suggest DAS-LHB may be comparable or noninferior.^48^, ^49^, ^50^ In their review of 51 shoulders, Garcia et al.^51^ observed an overall return-to-sport rate of 95.5% after remplissage but noted significant impairments in the throwing ability of overhead athletes. Patients reported pain with throwing, difficulties winding up, and stiffness. When differentiated by sport, rates of return were lowest at 50% for both baseball and basketball.^51^ In these subsets of patients, DAS-LHB’s preservation of motion may be advantageous and reduce risk of recurrent instability episodes. Future studies should examine use of DAS-LHB in these populations and consider supplementing with FBB procedures for additional stability if indicated.

### Limitations

This study had limitations that warrant discussion. First, a meta-analysis was not performed due to the included articles comprising a low level of evidence with differences in methodology. The indications for DAS-LHB are still being defined, and multiple variations in surgical technique are described in the literature. Second, differences in reported outcome measures limited the number of meaningful variables that could be examined. Third, the small number of studies discussing clinical outcomes precluded our ability to draw conclusions on how DAS-LHB compares to standard Bankart and Latarjet procedures in the long term. As discussed by Lӓdermann,^52^ the low rates of instability observed after DAS-LHB must be carefully contextualized. Studies reporting no recurrence or complications may have more adequate surgical indications or technique. Lastly, 1 study^31^ was deemed at high risk of bias and excluded from data analysis. This case series included clinical outcomes for DDP patients, a population that was observed to have the highest improvements in Rowe scores, FF, and ER. Inclusion of this article may have changed conclusions regarding this subset of patients.

## Conclusions

DAS-LHB in conjunction with Bankart repair preserves motion while showing clinically important functional improvements with low rates of recurrent instability or complication in the short-term postoperative period for patients with symptomatic shoulder instability.

## Disclosures

The authors declare the following financial interests/personal relationships which may be considered as potential competing interests: T.S. is a consultant or advisor for Stryker. B.W. is a board member of the American Academy of Orthopaedic Surgeons, American Orthopaedic Society for Sports Medicine, American Shoulder and Elbow Surgeons, Arthroscopy Association of North America, *Arthroscopy*, and *Video Journal of Sports Medicine*; has received speaking and lecture fees from Arthrex, Sparta, and Vericel; is a consultant or advisor for Arthrex; has equity or stocks with Kaliber AI, Sparta, and Vivorte; and has received funding grants from Smith & Nephew. All other authors (N.J., J.M., M.H., A.J.) declare that they have no known competing financial interests or personal relationships that could have appeared to influence the work reported in this paper.
