# Supplementary material for: Dynamic Anterior Shoulder Stabilization Using a Long Head of the Biceps Transfer and Bankart Repair Improves Clinical Outcomes in Patients With Subcritical Bone Loss: A Systematic Review
Source: Arthrosc Sports Med Rehabil. 2025 Apr 10;7(3):101141. doi: 10.1016/j.asmr.2025.101141 (PMC12276567; doi:10.1016/j.asmr.2025.101141)
Supplement: Supplementary Data [file mmc1.docx]

**Date of Search**: March 5^th^, 2025

**Databases used:** Pubmed, Scopus, Embase (Elsevier), Medline (Ovid)

**Search Strategy:**

Independent searches using the following key phrases were collated to ensure a complete collection of articles:

1. “dynamic anterior stabilization”
2. Dynamic anterior shoulder stabilization
3. Biceps transfer shoulder stabilization

Quotations in the first search enabled a focused search of articles, while subsequent key phrases without quotations broadened the results.

**Filters applied:**

**-**Studies published in the past ten years

-Studies published in the English language
